# Dupilumab in the treatment of severe atopic dermatitis refractory to systemic immunosuppression: case report

**DOI:** 10.31744/einstein_journal/2019RC4599

**Published:** 2019-06-28

**Authors:** Mara Huffenbaecher Giavina-Bianchi, Pedro Giavina-Bianchi, Luiz Vicente Rizzo

**Affiliations:** 1 Faculdade de Medicina, Universidade de São Paulo, São Paulo, SP, Brazil; 2 Hospital Israelita Albert Einstein, São Paulo, SP, Brazil

**Keywords:** Dermatitis, atopic/drug therapy, Immunosuppressive agents/therapeutic use, Severity of illness index, Dermatite atópica/tratamento farmacológico, Imunossupressores/uso terapêutico, Índice de gravidade de doença

## Abstract

Case report of a patient with severe atopic dermatitis who showed a good response to dupilumab. She had already used two immunosuppressive agents, cyclosporine A and mycophenolate mofetil, for the treatment of atopic dermatitis with no proper control of the disease. She had also been taking all measures to control severe cases of the disease: bath and environmental controls, topical potent corticosteroids and emollients. She presented constant pruritus and skin lesions, frequent skin infections e poor quality of life. She also developed depression due to her disease. Recently, dupilumab, a new biological agent, was approved for the treatment of moderate/severe atopic dermatitis in many countries, including Brazil. Dupilumab is a monoclonal antibody with a common alpha chain of interleukin (IL) 4 and IL-13 receptors, two cytokines involved in the Th2 profile immune response that promote atopic inflammation. In a pioneer way in Brazil, the patient initiated the treatment with an attack dose of 600mg subcutaneous of dupilumab and 300mg subcutaneous every other week. Up to now, she has taken four applications, presenting a great improvement of the disease and her quality of life. There were no adverse effects, nor in the injection site nor of other kind. Patient and her family are very satisfied, and the medical team evaluates that the treatment is being well succeed. The case report described here subsidizes the use of dupilumab in the treatment of severe atopic dermatitis refractory to use of immunosuppressive agents.

## RESUMO

Relatamos o caso de uma paciente com dermatite atópica grave, que mostrou boa resposta ao dupilumabe. Ela já tinha usado dois agentes imunossupressores, a ciclosporina A e o micofenolato de mofetila, para o tratamento da dermatite atópica, sem obter o controle adequado da doença. Ela também vinha fazendo uso de todas as medidas de controle para casos graves da doença: cuidados com o banho, controle ambiental, corticosteroides potentes tópicos e emolientes. Apresentava prurido e lesões cutâneas constantes, infeções de pele frequentes e qualidade de vida ruim. Passou a apresentar depressão devido à sua doença. Recentemente, o dupilumabe, um agente biológico novo, foi aprovado para o tratamento de dermatite atópica moderada a severa em muitos países, incluindo o Brasil. Dupilumabe é um anticorpo monoclonal cujo alvo é a cadeia alfa comum aos receptores da interleucina (IL) 4 e IL-13, duas citocinas envolvidas no perfil de resposta imune Th2, que promove inflamação atópica. De modo pioneiro no Brasil, a paciente iniciou o tratamento, com dose de ataque de 600mg por via subcutânea de dupilumabe e 300mg também por via subcutânea a cada 2 semanas. Até o momento deste relato, ela realizou quatro aplicações, apresentando grande melhora da doença e da qualidade de vida. Não houve efeitos adversos, nem no local da injeção e nem de outro tipo. A paciente e sua família estão muito satisfeitas, e os médicos que a tratam avaliam que a terapia está sendo bem-sucedida. Este relato de caso subsidia o uso de dupilumabe no tratamento da dermatite atópica grave refratária ao uso de imunossupressores.

## INTRODUCTION

Atopic dermatitis (AD) is a chronic, relapsing, inflammatory skin disease, with intense pruritus and erythematous or vesicular maculopapular lesions, with scaling, accompanied by dry skin, crusts and/or lichenification. Superinfection by viruses or bacteria is a frequent finding. Its prevalence, which is approximately 15% in children and 5% in adults, is increasing.^(^ ^1^ ^–^ ^3^ ^)^

Due to its chronic nature and frequent relapses, living with AD can be a burden, particularly for those requiring long-term, systemic treatment, since the drugs used can lead to serious toxicities. Pruritus and skin lesions can cause sleep disorders, anxiety, depression and low self-esteem, compromising the quality of life of patients and family.^(^ ^4^ ^)^

AD pathogenesis includes changes in skin barrier function, in some cases associated with mutations in the filaggrin gene, increased colonization by Staphylococcus aureus, and exacerbated Th2 immune response, with sensitization to allergens, increased IgE levels, and blood eosinophilia. The most frequently used immunosuppressants for AD are cyclosporine, mycophenolate mofetil, azathioprine, and methotrexate.^(^ ^5^ ^)^ New therapies based on AD pathogenesis have been developed, more effective and less detrimental, such as dupilumab, and will probably change the way we approach patients with moderate to severe AD.^(^ ^5^ ^)^

## CASE REPORT

A female, 18-year-old, Caucasian patient, born and residing in São Paulo. The patient has AD since she was 2 years old, with aggravation for the past 4 years, on 250mg cyclosporine (150mg in the morning and 100mg in the afternoon: 3.2mg/kg/day), 5mg desloratadine (morning), and 25mg hydroxyzine (evening), moisturizing lotion twice-daily, and clobetasol propionate cream twice-daily. History of allergic rhinitis, hypothyroidism and metabolic syndrome, on levothyroxine sodium 50mg/day and metformin hydrochloride 500mg/day.

The patient referred previous hospitalization for infection secondary to the skin lesions in late 2015, and five other subsequent episodes, treated in the outpatient setting. In November 2017, she was started on agomelatine 25mg/day and buspirone 5mg/day, due to depression and insomnia.

The disease remained poorly controlled, and the patient had very poor quality of life. After being on cyclosporine for more than 2 years uninterruptedly, without achieving proper control of the disease, the patient was started on mycophenolate mofetil, 1g every 12 hours, in the end of 2017. Cyclosporine was gradually tapered, and eventually discontinued. After switching medications, there were some side effects such as 12kg weight loss over 2 months, menstrual changes and telogen effluvium, but the regimen was maintained. However, with no clinical improvement.

The skin exam showed extensive eczema affecting 90% of the skin tissue, along with very intense pruritus and dry skin, and a Score for Atopic Dermatitis (SCORAD) of 45. The SCORAD is a tool to assess the severity of AD using a signs and symptoms scale ranging from zero (no lesions and symptoms) to 103 (maximum score). Over 40, AD is considered severe.^(^ ^6^ ^)^

**Figure 1:**
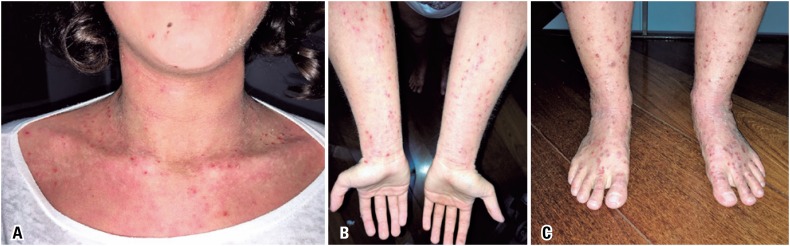
Atopic dermatitis lesions before dupilumab. (A) Lesions on the neck and upper torso, pre-treatment; (B) Lesions on upper limbs, pre-treatment; (C) Lesions on lower limbs, pre-treatment

After the dupilumab results were published and the monoclonal antibody was approved in different countries, including Brazil, it was indicated for this patient.^(^ ^5^ ^)^ In March 2018, the patient received her first dose of 600mg dupilumab, subcutaneously. The loading dose was followed by 300mg every other week. Mycophenolate was discontinued one month after the biological agent was introduced. In the fifth dose, the patient was already showing considerable improvement. She is currently on 25mg hydroxyzine every evening. Desloratadine, the topical corticosteroid and the antidepressants have been discontinued. The skin exam showed major improvements, as well as dry skin and pruritus. The current SCORAD is 16. Figures 1A to 1C (pre-treatment) and 2A to 2C (after three applications) show the level of improvement achieved between the first and third doses.

**Figure 2:**
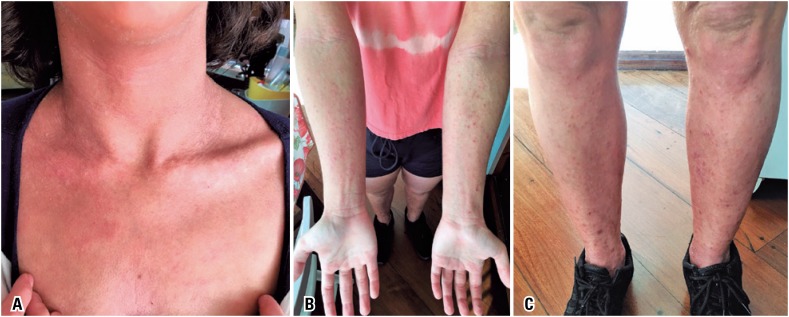
Atopic dermatitis lesions after dupilumab. (A) Lesions on the neck and upper torso after 3 applications; (B) Lesions on upper limbs after 3 applications; (C) Lesions on lower limbs after 3 applications

## DISCUSSION

We report the case of a patient with severe AD, poorly controlled, even with the most effective systemic therapies currently available in Brazil. The patient had used two systemic immunosuppressants, cyclosporine and mycophenolate mofetil, without achieving proper disease control. The patient had ongoing pruritus and skin lesions, in addition to greatly impaired quality of life, and depression.

Traditionally, cyclosporine is the first choice of treatment, since it is approved in many countries and has rapid onset of action. It is a calcineurin inhibitor, which inhibits interleukin (IL) 2 and T-cell activation, reducing immunoreactivity.^(^ ^7^ ^)^ The dose for adults is 3 to 5mg/kg/day, split in two (morning and evening). Mycophenolate mofetil is a prodrug of mycophenolic acid, derived from *Penicillium echinulatum*, and a metabolite that blocks T- and B-cell proliferation.^(^ ^8^ ^)^ It is used for severe cases of AD in children or adults who do not respond to cyclosporine.^(^ ^9^ ^)^ Its efficacy is comparable to that of cyclosporine and, despite its longer onset of action, results last longer. The dose ranges from 600 to 1.200mg/m^2^/day, or 40 to 50mg/kg/day in small children, 30 to 40mg/kg/day in adolescents, and 2g/day in adults.

Dupilumab is a fully humanized monoclonal antibody directly targeting the shared alpha chain of IL-4 and IL-13 receptors. These two cytokines are involved in Th2 immune response, inducing allergen sensitization, promoting atopic inflammation, and decreasing the skin barrier function and structure.^(^ ^10^ ^)^ The antibody inhibits the action of these cytokines, and has been associated with gene expression changes in AD lesions, improving their molecular signature.^(^ ^11^ ^)^ In a phase III clinical trial with 1,379 adult subjects with moderate to severe AD, poorly controlled with topical treatment, dupilumab was able to improve the signs and symptoms of the disease, including pruritus, anxiety, depression and quality of life. Skin infections were significantly less frequent in the treatment group *versus* placebo. The two regimens tested, 300mg subcutaneously every week or 300mg subcutaneously every other week for 16 weeks, were equally effective and safe. The most frequent side effects were injection site reactions and conjunctivitis.^(^ ^12^ ^)^ It is considered a breakthrough therapy for moderate to severe AD in poorly controlled adults. There are new studies in progress in children.

## CONCLUSION

We report the first case in Brazil using dupilumab, a new class of drugs for controlling atopic dermatitis, in a patient with severe disease, poorly controlled by commonly used systemic therapies, who, to date, is evolving quite well, with no adverse effects. This case report supports the use of dupilumab in treating severe atopic dermatitis, refractory to the use of systemic immunosuppressants.
